# Oligometastasis and local ablation in the era of systemic targeted and immunotherapy

**DOI:** 10.1186/s13014-020-01544-0

**Published:** 2020-05-04

**Authors:** Rosario Mazzola, Barbara Alicja Jereczek-Fossa, Davide Franceschini, Slavisa Tubin, Andrea Riccardo Filippi, Maria Tolia, Andrea Lancia, Giuseppe Minniti, Stefanie Corradini, Stefano Arcangeli, Marta Scorsetti, Filippo Alongi

**Affiliations:** 1 IRCCS, Advanced Radiation Oncology Department, Sacro Cuore Don Calabria Hospital, Negrar di Valpolicella, Verona, Italy; 2 Department of Radiation Oncology IEO, European Institute of Oncology IRCCS, Milan, Italy; 3 Department of Oncology and Hemato-oncology University of Milan, Milan, Italy; 4 Radiotherapy and Radiosurgery Department, Humanitas Cancer Center, Rozzano, Milan, Italy; 5 KABEG Klinikum Klagenfurt, Institute of Radiation Oncology, Klagenfurt am Wörthersee, Austria; 6 Radiation Oncology, Fondazione IRCCS Policlinico San Matteo, Pavia, Italy; 7 Faculty of Medicine, School of Health Sciences, University of Thessaly, University Hospital of Larissa, Biopolis, Larisa, Greece; 8 Radiation Oncology Unit, Department of Medicine, Surgery and Neuroscience, University of Siena, Siena, Italy; 9 Department of Radiation Oncology, University Hospital, LMU Munich, Munich, Germany; 10 Department of Radiation Oncology, Policlinico S. Gerardo and University of Milan “Bicocca”, Milan, Italy; 11 University of Brescia, Brescia, Italy

**Keywords:** Oligometastasis, Immunotherapy, Target therapy, Metastases-directed therapy

## Abstract

**Background:**

During these last years, new agents have dramatically improved the survival of the metastatic patients. Oligometastases represent a continuous field of interest in which the integration of metastases-directed therapy and drugs could further improve the oncologic outcomes.

Herein a narrative review is performed regarding the main rationale in combining immunotherapy and target therapies with SBRT looking at the available clinical data in case of oligometastatic NSCLC, Melanoma and Kidney cancer.

**Material and method:**

Narrative Review regarding retrospective and prospective studies published between January 2009 to November 2019 with at least 20 patients analyzed.

**Results:**

Concerning the combination between SBRT and Immunotherapy, the correct sequence of remains uncertain, and seems to be drug-dependent. The optimal patients’ selection is crucial to expect substantial benefits to SBRT/Immunotherapy combination and, among several factors. A potential field of interest is represented by the so-called oligoprogressed disease, in which SBRT could improve the long-term efficacy of the existing target therapy.

**Conclusions:**

A low tumor burden seems to be the most relevant, thus making the oligometastatic disease represent the ideal setting for the use of combination therapies with immunological drugs.

## Background

The term oligometastases is referred to a limited tumor burden potentially amenable to local approaches. In this last clinical scenario, high-dose radiation therapy, also known as stereotactic body radiotherapy (SBRT), represents a viable treatment option able to modify the natural history of the oligometastatic disease [1–5].

During these last years, new agents have dramatically improved the survival of metastatic patients. Melanoma, Kidney and NSCLC represent the oncologic diseases in which targeted drugs and/or immunotherapy are changing the daily clinical practice. The rationale in combining targeted agents and/or immunotherapy with SBRT could be to improve the therapeutic ratio through increased tumor cell killing while maintaining stable or decreased toxicity [6]. Compared to conventionally fractionated radiotherapy, SBRT induces direct tumor vascular-endothelial damage that may enhance the delivery of targeted agents to the tumor [7–9]. All these effects seem to be SBRT-related appearing exclusively using larger fraction-doses, while are not found with conventionally fractionated radiotherapy. Targeting agents are directed against specific molecular mutations, aberrant intracellular signaling or repair pathways, negatively affecting carcinogenesis and tumor growth. If combined with radiation, these “smart drugs” might boost tumor responses to SBRT through those specific mechanisms they exert. Example of a highly rational combination would be synergistic anti-angiogenic effects of bevacizumab, which inhibits the development of tumor vasculature by targeting VEGF, and fulminant tumor vascular-endothelial damage induced by SBRT.

A combination of radio-immunotherapy originates from the significant immune-stimulatory effects they both exert boosting the natural antitumor immune response through synergistic potentiation of an immunomodulatory effect, possibly leading to an abscopal effect. This radiation-induced immune-mediated but rare systemic antitumor phenomenon that has high therapeutic potential, is more probable if induced by SBRT associated to checkpoint inhibitors [10, 11]. SBRT, through released neo-antigens and consequent maturation and proliferation of naive T-cells, and immunotherapy through activation and amplification of naive T-cells, may reciprocally potentiate each other amplification of T-cells-mediated tumoricidal effects (mixed synergistic-additive effects) [12–14]. The lack of evidence prevents us from understanding which would be the finest time-sequencing of radio-immunotherapy, and which radiation dose-fractionation would be most “immunogenic”. It seems that concurrent treatment or close sequencing of immunotherapy following radiotherapy may take the most immunogenic advantage [12]. While the radiation dose required for the maximum local tumor-control has to be the highest reasonably achievable, for the strongest antitumor immune response should not necessarily be that high, but rather a sub-tumoricidal dose. Several preclinical studies suggested doses 8 to 10Gy per fraction in 1–3 fractions to be optimally immunogenic [12–14].

Herein a narrative review is performed regarding the main rationale in combining immunotherapy and target therapies with SBRT looking at the available clinical data in case of oligometastatic NSCLC, melanoma and kidney cancers.

Retrospective and prospective studies published between January 2009 to November 2019 with at least 20 patients were analyzed.

### Oligometastatc non-small-cell lung cancer

#### Immunotherapy and high doses RT

Most of the available data are retrospective experiences on patients with brain oligorecurrence treated with radiosurgery (SRS) or hypofractionated RT. Chen et al. analyzed 37 NSCLC patients treated with SRS for brain metastases in combination with different checkpoint inhibitors. Data on these patients are enclosed in a larger series analyzing also patients with melanoma and renal cancer. Interestingly, the authors demonstrated that patients receiving RT concomitantly with immunotherapy had a longer OS (24.7 months) if compared with patients receiving both treatments but not concurrently (14.5 months). No increased rates of immune-related adverse events or acute neurologic toxicity were reported [15]. No safety concerns came also from a recent study by Verma et al. of thoracic RT combined with immunotherapy. In the 60 patients treated with 50 Gy/4 fractions or 60 Gy/10 fractions and concurrent Ipilimumab or Pembrolizumab, no patient experienced grade 4 adverse events, while 34 grade 3 events in a total of 15 patients were recorded. No difference in terms of toxicity was detected in patients receiving pembrolizumab or ipilimumab. Efficacy data are pending [16].

Concerning prospective data, two phase II trials combining RT and IT were recently published.

The Pembro-RT trial [17] enrolled NSCLC patients with at least 2 metastases (upper limit was not specified). Patients were randomized to receive Pembrolizumab or Pembrolizumab + SBRT to a single metastatic site, in order to increase the likelihood of abscopal effect. The dose chosen for SBRT was 24 Gy in 3 fractions, based on preclinical data suggesting that this schedule could increase the synergism between RT and the immune system [18]. The primary endpoint of the study was not reached, however experimental arm performed better than control arm for all endpoints. Objective response rate at 12 weeks was doubled (36% vs 18%), median PFS and OS were also improved (6.6 months and 15.9 months respectively). Addition of SBRT to Pembrolizumab did not increase toxicity.

Bauml et al. [19] conducted a single arm phase II trial specifically focused on oligometastatic NSCLC (less than 4 metastatic sites) patients treated with local ablative therapies (including SBRT in 30 patients) on all sites and Pembrolizumab. Median PFS from local therapy was 19.1 months and median PFS from starting of Pembrolizumab was 18.7 months. Both results were significantly better than the historical control reporting a PFS of 6.6 months. Overall survival rate at 12 and 24 months was 90.9 and 77.5%. Again, no safety concern emerged.

A summary of the main studies combining high dose RT and immunotherapy is reported in Tables 1 and 2.

**Table 1:** Patients and tumor characteristic of the selected studies

**Authors** **(year of publication)** **[Reference]**	**Number of patients underwent SBRT**	**Type of Study**	**Primary Tumor site**	**Number of metastases underwent to SBRT (upper limit)**	**Type of oligometastases and organ involved (liver, lung, bone, nodes, brain…)**	**Metastases size** **(cm)**	**Median total dose/fraction**	**Biologically equivalent dose (Median value)**^**a**^	**Systemic Therapy**
Theelen et al. (2019) [17]	35	Phase II	NSCLC	1	Immunostimulation (Lung, Nodes, Adrenal, Bone, Skin, Liver, Pleura)	N.S.	24/3	43.2	Pembrolizumab
Lesueur et al. (2018) [20]	28	Retrospective	NSCLC	1	Oligorecurrent (Bone, Brain, Lung)	N.S.	25–30/1–3	81.6/60	Nivolumab
Chen et al. (2018) [15]	37	Retrospective	NSCLC	2	Olicorecurrent (Brain)	N.S.	24–24-25/1–3-5	81.6/38.4/37.5	Ipilimumab/Nivolumab/Pembrolizumab
Schapira et al. (2017) [21]	37	Retrospective	NSCLC	7	Oligorecurrent (Brain)	0.6	18–17-21/1–1-3	50.4/45.9/35.7	Nivolumab/Atezolizumab/Pembrolizumab
Bauml et al. (2019) [19]	45	Phase II	NSCLC	4	Oligoprogressive (N.S.)	N.S.	N.S.	N.S.	Pembrolizumab
Hubbeling et al. (2018) [22]	35	Retrospective	NSCLC	10	Oligoprogressive (Brain)	N.S.	N.S.	N.S.	Nivolumab, Atezolizumab, Pembrolizumab
Verma et al. (2018) [16]	41	Retrospective	NSCLC	N.S.	Oligoprogressive (Extracranial)	N.S.	50/4	112,5	Pembrolizumab

^a^Alpha/Beta 10; *N.S.* Not specified

**Table 2:** Clinical outcomes by the selected studies

**Authors** **(year of publication)**	**Local Control**	**Distant progression free survival**	**Overall Survival**	**Toxicity**
Theelen et al. (2019) [17]	NS	6.6 months	15.9 months	12 > G3
Lesueur et al. (2018) [20]	64,4% 2 yr	2,7 months	11,1 months	14,4% > G3
Chen et al. (2018) [15]	88% 1 yr	2.3 months	24,7 months	16% > G3
Schapira et al. (2017) [21]	100% 1 yr	N.S.	17.6 months	0 ≥ G4
Bauml et al. (2019) [19]	N.S.	19.1 months	41.6 months	5 > G3
Hubbeling et al. (2018) [22]	N.S.	N.S.	N.S.	9 > G3
Verma et al. (2018) [16]	N.S.	N.S.	N.S.	25 > G3

#### Target therapy and high doses RT

A phase II study enrolled 24 unselected NSCLC patients with six or fewer sites of extracranial progression after first line chemotherapy. All were then treated with Erlotinib and SBRT, obtaining a median PFS and OS of 14.7 and 20.4 months. Upon progression, only three of 47 measurable lesions recurred within the SBRT field [23].

Qiu et al. analyzed data from 46 patients, treated with local therapies (all but two with RT) and continuing the same TKI. Twenty-four (52.2%) patients were treated for brain metastases, 16 (34.8%) patients for lung metastases, and 6 patients for bone metastases. The median overall and progression-free survival after the local treatment were 13.0 and 7.0 months, respectively. The 2-year OS was 65.2% [24].

Borghetti et al. analyzed 106 patients treated with RT concomitant to TKIs (EGFR or ALK inhibitors). Almost half of these patients were defined as oligometastatic/oligoprogressive patients. Sites of RT were brain, bone, lung or others in 46, 27, 14 and 13%, respectively. OS at 1 and 2 years in oligometastatic/oligoprogressive patients were 79 and 61.8%, respectively [25].

Rossi et al. reported on 131 patients experiencing disease progression during first-line Afatinib or Gefitinib. Thirty of these patients received local therapy with high dose RT and continued the same drug. Median overall survival resulted longer in these patients when compared with patients continuing TT beyond progression or patients switching to another systemic therapy (*p* < 0.0001). There was also a trend towards a longer second progression-free survival (measured from the time of first progression until second progression) (*p* = 0.06) [26]. A different approach has been studied by Xu and colleagues. They anticipated the local consolidation in oncogene driven NSCLC patients after few months of TKI, without waiting for the unavoidable progression. Patients were divided into 3 groups: 51 patients received consolidative therapy to all residual disease, 55 patients received consolidative therapy to either primary tumor or oligometastatic sites, while 39 patients did not receive any local treatment. The median PFS was improved in the first group when compared to other groups, 20.6, 15.6, and 13.9 months, respectively (*P* < 0.001). The median OS were 40.9, 34.1, and 30.8 months in the three groups, respectively (P < 0.001). Of note, the difference was statistically significant between patients treated to all residual disease, while it was not significant between patients who receive a partial local treatment and patients who did not receive any local therapy at all [27].

Finally, concerning the safety profile of the combination between EGFR or ALK inhibitor and high dose RT, none of the available studies showed a significant increase in side effects [28].

A summary of the main studies combining high dose RT and Target Therapy is reported in Tables 3 and 4.

**Table 3:** Patients and tumor characteristics of the selected studies

**Authors** **(year of publication) [reference]**	**Number of patients underwent SBRT**	**Type of Study**	**Primary Tumor site**	**Number of metastases underwent to SBRT (upper limit)**	**Type of oligometastases and organ involved (liver, lung, bone, nodes, brain…)**	**Metastases size** **(cm)**	**SBRT** **(median total dose/fraction)**	**Biologically equivalent dose (Median value)**	**Systemic Therapy**
Weickhardt et al. (2012) [29]	25	Retrospective	NSCLC	<=4	Oligoprogressive (Brain,Lung)	N.S.	15–54Gy, median 40Gy	N.S.	Crizotinib, Erlotinib
Iyengar et al. (2014) [23]	24 (52 lesions)	Phase II	NSCLC	<=3	Oligorecurrent (Lung; Liver; Kidney; Bone; Adrenal; Mediastinum)	N.S.	19–40/1–5	55.1–72	Erlotinib
Borghetti et al. (2019) [25]	49	Retrospective	NSCLC	<=4	Oligoprogressive (Brain, Lung, Bone)	N.S.	mean 80 Gy, range 60–178 Gy	> 60	N.S.
Qiu et al. (2017) [24]	46	Retrospective	NSCLC	< 5	Oligoprogressive	N.S.	N.S.	N.S.	N.S.
Rossi et al. (2019) [26]	30	Retrospective	NSCLC	N.S.	Oligoprogressive	N.S.	N.S.	N.S.	Afatinib, Gefitinib
Weiss et al. (2019) [30]	25	Retrospective	NSCLC	N.S.	Oligoprogressive	N.S.	N.S.	N.S.	Erlotinib
Chan OSH et al. (2018) [31]	18	Phase II	NSCLC	34	Oligoprogressive	N.S.	N.S.	N.S.	TKI therapy
Xu et al. (2018) [27]	51	Retrospective	NSCLC	N.S.	Oligoprogressive	N.S.	27–21–33-37.5/1–1–3-5	65.8	Gefitinib, Erlotinib, Icotinib

**Table 4:** Clinical outcomes by the selected studies

**Authors** **(year of publication) [reference]**	**Local Control**	**Distant progression free survival**	**Overall Survival**	**Toxicity**
Weickhardt et al. (2012) [29]	N.S.	6.2 months	N.S.	2 ≥ G3
Iyengar et al. (2014) [23]	N.S.	14.7 months	20.4 months	2 > G3
Borghetti et al. (2019) [25]	N.S.	N.S.	23 months	0 > G3
Qiu et al. (2017) [24]	81.4%	7 months	35 months	2 > G3
Rossi et al. (2019) [26]	N.S.	13.8 months	35 months	N.S.
Weiss et al. (2019) [30]	N.S.	6 months	29 months	N.S.
Chan OSH et al. (2018) [31]	N.S.	15 months	N.S.	0 > G3
Xu et al. (2018) [27]	N.S.	20.6 months	40.9 months	14% > G3

### Oligometastatic melanoma

#### Immunotherapy and high doses RT

Different retrospective studies have demonstrated an OS and/or intracranial control benefit of immune checkpoint inhibitors when used in combination with SRS for the treatment of melanoma brain metastases. In their mono-institutional analysis, Qin and colleagues [32] found a trend toward improved OS in advanced melanoma patients receiving Ipilimumab and ablative radiotherapy. An increased response duration was observed when RT was delivered after immunotherapy, while toxicity rates did not undergo substantial changes.

A large retrospective analysis made at Johns Hopskins hospital [15] and including patients diagnosed with brain metastases from different primary tumors, who underwent SRS with and without concurrent therapy with Immunotherapy, found a lower incidence of new intracranial metastases in those who received the combined treatment, with favorable survival outcomes and limited side effects. These last results are consistent with the ones from Diao et colleagues [33], which found a substantial improvement in median OS for patients with brain metastases treated with SRS and Ipilimumab. Four cases (17%) of acute neurologic toxicity > G2 and 4 cases (17%) of late radiation necrosis were reported.

The association between SRS and the anti-PD-1 Pembrolizumab also showed its efficacy in a retrospective study from MSKCC [34], with a marked reduction in the size of melanoma brain metastases at the time of first follow-up.

Concerning extracranial disease localization, Gabani et al. [35] found that the addition of SBRT to immunotherapy in an unselected patient population does not seem to be beneficial if compared with immunotherapy alone. More specifically, irradiation to bone metastasis was found to be associated with worse OS than those treated with Immunotherapy alone. The only significant association with improved OS was found for patients who received early SBRT to soft tissue metastases (at least 30 days before starting immunotherapy).

A summary of the main studies combining high dose RT and immunotherapy is reported in Tables 5 and 6.

**Table 5:** Patients and tumor characteristics of the selected studies Melanoma and Immunotherapy

**Authors** **(year of publication)** **[reference]**	**N of pts underwent SBRT**	**Type of study**	**Primary tumor**	**N of mts underwent to SBRT**	**Type of Oligometases and organ involved**	**Median total dose per fraction**	**BED**	**Systemic Therapy**	**Mts size (cm),** **median**
Gabani 2018 [35]	77 (288 received RT generically)	retrospective	Melanoma		Extracranial (bone, soft tissues, lung..)	30 Gy (5fx)		Ipi, Pembro, Nivo, Il-2, Vaccines	**N/A**
Stera 2018 [36]	48^a^ (35 received ICI)	retrospective	Melanoma	250	Brain, Extracranial (32pts.)	18 Gy	BED10 50.4 Gy	ICI, BRAFi	0.23 cm3 (per lesion)
Liniker 2016 [37]	35	retrospective	Melanoma		Brain or Extracranial			Anti-PD1	
Qin 2015 [32]	21	retrospective	Melanoma	N.S.	Brain			Ipi	
Diao 2018 [33]	51^b^	retrospective	Melanoma	155	Brain	20 Gy		Ipi	0.27 cm3
Anderson 2017 [34]	18 (11 SRS + 7 hypoRT)	retrospective	Melanoma	23	Brain	20 Gy (1) < 2 cm; 18 Gy(1) < 3 cm; 30Gy(5) > 3 cm		Pembro	1 cm (SRS)
Chen 2017 [15]	260 (70 melanoma pts.^d^.)	retrospective	NSCLC, RCC, Melanoma	623 (total)	Brain	20 Gy		Anti PD-1, Anti Ctla4	
Chandra 2015 [38]	47^c^	retrospective	melanoma	18	Brain	20Gy			4 cm

^a^including also pts. treated with BRAFi

^b^23 concurrently, 28 sequentially

^c^including pts. receiving non SRS/RT

^d^including those treated with WBRT

**Table 6:** Clinical outcomes by the selected studies Melanoma and Immunotherapy

**Authors (year of publication)** **[reference]**	**Local Control**	**Progression free survival**	**Overall Survival**	**Toxicity**
Gabani 2018 [35]	N/A	N/A	15.4 mo (median)	N.S.
Stera 2018 [36]	1 yr LCR: 89.5%	6 mo: 42.3% 1 yr: 25.5%	6 mo: 75.3% 1 yr: 50.8% 2 yr: 31.8%	3 > G2 (1 autoimmune hypophysitis, 1 autoimmune pancreatitis, 1 radionecrosis)^a^
Liniker 2016 [37]	RR: 44% e 64% ^b^			3 > G2 (1 case of radiation necrosis, 2 radiation dermatitis)
Qin 2015 [32]	Ipi before RT > 6 and 12 mo response duration that Ipi after RT		19.6 mo (median) 6 mo: 95.1% 1 yr: 79.7%	Pts. Who received Ipi after radiation had fewer irAEs than those who received it before radiation
Diao 2018 [33]	Non-concurrent Ipi: 1 yr, 70% Concurrent Ipi: 1 yr, 58%	N.S.	Non-concurrent Ipi: Median,18.7 mo 1 yr, 63% Concurrent Ipi Median, 11.8 mo 1 yr, 50%	Acute 4 > G2 (2 cases of cerebral oedema, 2 cases of cerebral hemorrhage)^c^ Late 4 > G2 (Radiation Necrosis) No G5 events
Anderson 2017 [34]	93% (at the time of death)	N.S.	N.S.	No > G3 events 1 G2 CNS bleeding
Chen 2017 [15]	Non concurrent ICI: 1 yr 79% Concurrent ICI: 1 yr 88%	N.S.	Concurrent ICI: 24.7 mo Non-Concurrent ICI: 14.5 mo	3% G3 acute CNS No > G3 events
Chandra 2015 [38]		N.S.	28 mo (median)	N.S.

^a^only attributable to SRS/SBRT+Immunotherapy

^b^44% response rate for lesions treated sequentially, 64% for lesions treated concurrently

^c^2/4 side effects reported in pts. who did not receive Immunotherapy

#### Target therapy and high-dose RT

Wolf and colleagues [39] reported the results of one of the first prospective experiences on the association of SRS with BRAF inhibitors in the treatment of melanoma patients who developed brain metastases. Overall survival was increased in patients harboring BRAF mutation (who received both therapies) compared to BRAF-wild type patients. The combined therapy was found to be safe, with no difference in terms of intracranial hemorrhage events between patients who were treated with systemic agent and those who also received SRS.

Several retrospective experiences on the combo SRS-target therapy were reported in the recent years. In their institutional analysis, Ahmed et al. [40] describe the outcomes of melanoma brain metastases treated with SRS and various systemic and targeted agents. Patients who received BFAF/MEK inhibitors or anti-PD-1/anti-CTLA-4 therapies had improved OS over patients who were treated with conventional chemotherapy on multivariate analysis from the date of SRS; significant difference was also noted on the rate of distant metastases control.

Gaudy Marqueste [41] provided other insights on the safety of the association between SRS and BRAF inhibitors. According to their analysis, the authors suggest not to withhold concomitant administration of Vemurafenib or Dabrafenib during SRS, while this precaution can still be valid in the case of other radiotherapy techniques, including Whole Brain Radiotherapy (WBRT), which implicate larger areas of healthy brain irradiation.

However, these data are not concordant with those reported by other authors. In fact, increased hemorrhage risk was noted by Ly et al. [42] in a subgroup of melanoma patients metastatic to the brain who received SRS together with BRAF inhibitors, despite the improved local control rates. Patel et al. noticed higher rates of both symptomatic and radiographic radiation necrosis in the same setting of patients [43].

We currently have fewer data concerning the role of SBRT in patients diagnosed with extracranial metastatic melanoma and undergoing BRAF inhibitors. Franceschini et al. [44] have reported that such therapeutic strategy is feasible and well tolerated, even though survival outcomes remain insufficient; however, LC of the irradiated lesions showed a significant impact on OS.

A summary of the main studies combining high dose RT and Target Therapy is reported in Tables 7a, b.

**Table 7:** Patients and tumor characteristics of the selected studies Melanoma and Target Therapy

**a**									
**Authors** **(year of publication)** **[reference]**	**N of pts underwent SBRT**	**Type of study**	**Primary tumor**	**N of mts underwent to SBRT**	**Type of Oligometases and organ involved**	**Median total dose (dose per fraction**	**BED**	**Systemic Therapy**	**Mts size (cm)**
Kotecha 2017 [45]	191 (19 pts. had BRAF mutated tumors^a^)	retrospective	melanoma	793 (81 received BRAFi)	Brain	According to the RTOG protocol 90–05		BRAFi	1
Wolf 2016 [39]	80 (31 received BRAFi)	prospective	melanoma		Brain				
Franceschini 2017 [44]	31 (3 received BRAFi)	retrospective	melanoma	38	Extracranial (lung, liver, nodes)	48 Gy (4)	>100Gy in 74% of pts	BRAFi	39.6 cm3 (mean)
Ahmed 2016 [40]	96 (18 received BRAFi, 12 received BRAF/ MEKi)	retrospective	melanoma	314 (103 received Targeted Therapy)	Brain	24 Gy(1)		BRAFi or BRAF/ MEKi	0.1 cm3
Gaudy-Marqueste 2014 [41]	30	retrospective	melanoma	263	Brain	Range 20–28 Gy		Vemurafenib (26) or Dabrafenib (4)	N/A
Hecht 2018 [46]	39	retrospective	melanoma		Brain			Vemurafenib (23) or Dabrafenib (16)	
Ly 2015 [42]	52 (17 received BRAFi)	retrospective	melanoma	198 (96 received BRAFi)	Brain	20 Gy (1)			
Mastorakos 2018 [47]	67	retrospective	melanoma		Brain, extracranial mts.	According to RTOG 95–08 guidelines_ 19.2 Gy			1.1 cm3
Patel 2017 [43]	87 (15 received BRAFi)	retrospective	melanoma	157 (32 received BRAFi)	Brain	21 Gy		Vemurafenib (14) or Dabrafenib (1)	0.12 cm3
**b**									
**Authors (Year of Publication)** **[reference]**		**Local Control**		**Progression Free Survival**	**Overall Survival**			**Toxicity**	
Kotecha 2017 [45]		N.S.		N.S.	N.S.			N.S.	
Wolf 2016 [39]		94.6%		3.9 mo	13 mo (median)^a^ Actuarial: 83% at 3 mo 65% at 6 mo 46% at 12 mo			No increase in hemorrage rates in SRS + BRAFi	
Franceschini 2017 [44]		96.6% at 12 mo 82.8% at 24 mo		5.8 mo (median) Actuarial: 48.2% at 6 mo 18.5% at 12 mo 13.9% at 24 mo	10.6 mo (median) Actuarial: 77% at 6 mo 41% at 12 mo 21% at 24 mo			Acute 1 G2 (pneumonia) Late 1 G2 pneumonia 1 G2 dyspnea 1 gastric ulceration	
Ahmed 2016 [40]		89% at 6 mo 83% at 12 mo		3.4 mo (median) Actuarial(BRAF/MEKi) 58% at 6 mo 39% 1 t 12 mo Actuarial (BRAFi) 29% at 6 mo 12% at 12 mo	Actuarial (BRAF/MEKi) 83% at 6 mo 75% at 12 mo Actuarial (BRAFi) 71% at 6 mo 29% at 12 mo			1 G2 headache 1 radionecrosis	
Gaudy-Marqueste 2014 [41]					24.8 weeks (median)			20% of pts. presented neurological symptoms	
Hecht 2018 [46]		No difference in median values between Concomitant and Interrupted treatment		Concomitant BRAFi: 4.2 mo Interrupted BRAFi: 5.8 mo	Concomitant BRAFi: 7.3 mo Interrupted BRAFi: 9.8 mo			Radiation dermatits > G1: Concomitant BRAFI: 35% of pts. Interrupted BRAFi: 14%.	
Ly 2015 [42]		85% at 1 yr		32.3% at 1 yr	50.2% at 1 yr			Freedom from intratumoral hemmorrhage at 1 year: 39.3%	
Mastorakos 2018 [47]		23% local progression rate at 1 yr			13 mo (median) Actuarial: 70.1% at 6 mo 52.2% at 12 mo 20.9% at 24 mo			10.4% of pts. with Inttracranial hemmorhage	
Patel 2017 [43]		3.3% LR at 1 yr		N.S.	78.6% at 6 mo 64.3% at 12 mo			8 pts. developed symptomatic RN	

^a^from date of diagnosis of BM

### Oligometastatic renal cell carcinoma (mRCC)

#### Immunotherapy and high doses RT

Clinical evidences reporting on the combination of SBRT with Immunotherapy in mRCC are poor. Xie et al. [48] showed a systemic complete response to SBRT and Pembrolizumab in a patient affected by mRCC. SBRT consisted in the administration of 4 consecutive fractions up to a total dose of 32 Gy to a mediastinal enlarged lymph node compressing the esophagus. Matsushita et al. [49] recently reported on two patients with mRCC who received Nivolumab combined with external irradiation and obtained a marked reduction of metastatic diseases, including non-irradiated lesions, after being refractory to prior treatment with multiple targeted agents. Taken together, these experiences could suggest that it might be worthwhile to consider the addition of SBRT for oligometastatic RCC patients treated with checkpoint inhibitors, due to the additive or synergistic effects of this combination.

#### Target therapy and high doses RT

The available experiences regarding target therapies and high doses RT included both brain and extracranial mRCC. Cochran et al. [50] demonstrated a better local control for combined approach when compared to local therapy without targeted agents. In fact, the 1-year local control was 93.3 and 60% for patients treated with and without targeted agents, respectively. Contrarily, Verma et al. [51] has observed no improvement of local control with TKIs added to local brain therapy (surgery, SRS). Different patient populations across the studies [50, 51] (patients with brain metastases at relapse in the Verma series), well reflected in very different median survival rates (5.4 and 16.6 months, in Verma and Cochran series, respectively), might at least partially explain these contradictory results.

The studies on extracranial mRCC are not conclusive about the potential benefit of adding SRT to target therapy. In a recent phase I/II study including 13 patients treated with Pazopanib and SBRT local control and response rates outside the radiation field were good but seemed not to be superior when compared to SBRT or Pazopanib in monotherapy [52]. Contrarily, Dengina et al. [53] observed in a small phase 1b Volga Study (VEGFR inhibitor or mTOR inhibitor or checkpoint inhibitors and SRT) that the difference in response in the target and control metastases evaluated by a mean size of the lesions before and at 2 months after SBRT was statistically significant (*p* < 0.01). Miller et al. [54] demonstrated in the multivariate competing risks regression that concurrent first-line TKI treatment was independently associated with a local control benefit (HR 0.21, *p* = 0.04), while patients treated with TKIs alone experienced an increased rate of local failure (HR 2.43, *p* = 0.03). Franzese et al. [55] showed in univariate and multivariable analyses that metachronous and single metastasis but non addition of target therapy predicted better progression-free survival. However, when the analysis was restricted to cells clear RCC cases only, target therapy performed before SBRT improved local control (HR 0.15, 95% CI 0.026–0.085, *p* = 0.032), suggesting different biological response of cell clear RCC to the combination of SBRT and targeted agents.

There are several ongoing or just completed prospective studies on SBRT for oligometastatic RCC [56].

A summary of the main studies combining high dose RT and Target Therapy is reported in Tables 8 and 9.

**Table 8:** Patients and tumor caracteristics of the selected studies Kidney and target therapy

**Authors** **(year of publication)** **[reference]**	**Number of patients underwent SBRT**	**Type of Study**	**Primary Tumor site**	**Number of metastases underwent to SBRT**	**Type of oligometastases and organ involved (liver, lung, bone, nodes, brain…)**	**Metastases size** **(cm)**	**Median total dose/fraction**	**BED (Median value)**	**Systemic Therapy**
Staehler 2011 [57]	106	Retrospective	Kidney	N.S.	Spinal (55 pts) Brain (51 pts)	in cm^3^ spinal: 30.1 brain: 1.7	20 Gy in single fraction	N.S.	Sunitinib (45 pts) Sorafenib (61 pts)
Staehler 2012 [58]	22	Prospective	Kidney	N.S.	Progressive RCC in brain, retroperitoneal mediastinal lymph nodes, spinal cord, bones, liver, and kidney	N.S.	40 Gy in 8 fr (5Gy/fraction)^a^	N.S.	Sunitinib
Cochran 2012 [50]	61 (24 pts. received target therapy)	Retrospective	Kidney	N.S.	Brain	N.S.	20 Gy in single fraction	N.S.	TKIs, mTORIs, or bevacizumab (24 pts)
Verma 2013 [51]	81 (40 pts. received target therapy)	Retrospective	Kidney	216	Brain (at diagnosis and at relapse)	N.S.	SRS in 89 lesions^c^	N.S.	TKIs (40 pts)
Bastos 2015 [59]	65	Retrospective	Kidney	SRT^b^ (41 pts)	Brain (54% of pts. more than 1 met)	N.S.	N.S.	N.S.	antiangiogenetic (anti-VEGF, temsirolimus, sorafenib, bevacizumab, everolimus, pazopanib, axitinib) (53 pts) mTORIs (12 pts)
Miller 2016 [54]	100 (46 pts. received target therapy)	Retrospective	Kidney	N.S.	Spine	N.S.	16 Gy in 1 fraction	N.S.	TKI
Franzese 2019 [55]	58 (38 pts. received target therapy)	Retrospective	Kidney	73	Extracranial oligometastases	2.6 cm (diameter)	45 Gy in 5 fractions (9Gy/fraction)	N.S.	TKI or “other target therapies” (28 pts. received therapy before and 17 pts. – during SRT)

Legend:

*Met* Metastasis

*mTORIs* Mammalian target of rapamycin inhibitors

*NS.* Not specified

*Pts* Patients

*RCC* Renal cell carcinoma

*SRS* Stereotactic radiosurgery

*TKIs* Tyrosine kinase inhibitors

*VEGF* Vascular endothelial growth factor

Notes:

^a^some received moderately hypofractionated RT schedules

^b^RT was administered before systemic therapy (time interval is unknown)

^c^other patients received surgery, whole brain radiotherapy o no local brain treatment

Type of Study: Prospective, retrospective, Randomized…

Type of oligometastases: oligorecurrent, oligoprogressive, oligopersistent…

Systemic Therapy: which drug?

**Table 9:** Clinical outcomes by the selected studies Kidney and target therapy

**Authors** **(year of publication)** **[reference]**	**Local Control**	**Distant progression free survival**	**Overall Survival**	**Toxicity**
Staehler 2011 [57]	98% at 15 months	N.S.	17.4 months (spinal lesions) 11.1 months (brain lesions)	2 pts.: asymptomatic tumour haemorrhage after SRS (G2) 3 pts.: convulsions (G2)
Staehler 2012 [58]	NS (1 case of PD at first evaluation at 3 months, other patients remission or stable disease)	N.S.	65% at 2 years	No G3 during combination
Cochran 2012 [50]	74% at 1 year 40% at 3 years (better for combined therapy: 1 year LC was 93.3 and 60% for patients treated with and without targeted agents, respectively)	N.S.	38% at 1 year, 9% at 3 years (median survival 16.6 months for pts. treated with target therapy)	6 pts.: brain edema or necrosis (3 of them received target therapy) 2 brain hemorrhage
Verma 2013 [51]	75.6% at 1 year in pts. treated with SRS LC was statistically superior in lesions managed with surgery or SRS vs. the no local therapy. No improvement of LC with TKIs added to local therapy (surgery, SRS)	N.S.	5.4 months (all pts)	4 pts.: radionecrosis (2 of them in the TKI group and 2 in the non-TKI group)
Bastos 2015 [59]	N.S.	N.S.	12.2 months	5 pts. (8%): neurological 2 pts.: brain necrosis 3 pts.: brain hemorrhage
Miller 2016 [54]	Subgroup SRS + TKI: 94% at 1 year	N.S.	N.S.	No G3 in TKI + SRS pts., the incidence of post-SRS vertebral fracture (overall 21%) and pain flare (overall 17%) were similar across cohorts (TKI, SRS alone, TKI + SRS)
Franzese 2019 [55]	90.2% at 1 and 1.5 year	N.S.	100% at 1 year 83% at 5 years	No G3 acute or late toxicity

Notes and legend:

*LC* Local control

*NS* Not specified

*PD* Progressive disease

*Pts* Patients

*SRS* Stereotactic radiosurgery (single fraction)

*TKIs* Tyrosine kinase inhibitors

## Conclusions

The therapeutic scenario of oligometastatic diseases has dramatically changed during the recent years, thanks to the introduction of the so-called metastases-directed therapy (SBRT) in combination with standard of care drugs [60]. The scientific community has focusing own interest to explore the possibility to combine new agents with SBRT to improve the therapeutic window.

Concerning the combination between SBRT and Immunotherapy, the correct sequence of remains uncertain, and seems to be drug-dependent: best results were seen when CTLA-4 was given before SBRT while inhibition of the PD-1 axis has been proved to be most efficient when given in close temporal relation to the radiation treatment. Secondly, SBRT should be carefully taken into account as the most currently employed such as intensity modulated radiotherapy leads to a low-dose bath to a large part of the body, thus potentially interfering with the priming process of T lymphocytes – the most radiosensitive cells in the body – and its memory functions. Last, optimal patients’ selection is crucial to expect substantial benefits to SBRT/Immunotherapy combination and, among several factors, a low tumor burden seems to be the most relevant, thus making the oligometastatic disease the ideal setting for the use of combination therapies with immunological drugs.

Regarding target therapy and SBRT a field of interest is represented by the so-called oligoprogressed disease during targeted therapies. In fact, it is common to observe isolated disease progression in few sites, usually one to three, in a scenario of disease controlled by systemic therapy. In this last clinical scenario, the main aim of SBRT is the prolongation of efficacy of the existing target therapy, the delay of the switch to other systemic therapies and the improvement of patients’outcome modifying the natural history of the disease.

In the setting of oligometastatic disease, the combination of these new drugs with ablative doses of RT to limited tumor sites has brought a momentous improvement in disease control rates.

## Data Availability

Not applicable.

## Abbreviations

OAR: Organs at risk
SBRT: Stereotactic body radiation therapy
VEGF: Vascular endothelial growth factor
AE: Abscopal effect
CTLA-4: Cytotoxic T-lymphocyte-associated Protein 4
PD-1: Programmed cell death protein 1
HR: Hazard ratio
mTORIs: Mammalian target of rapamycin inhibitors
RCC: Renal cell carcinoma
RT: Radiotherapy
SBRT: Stereotactic radiotherapy
SRS: Stereotactic radiosurgery (single fraction SRT)
TKIs: Tyrosine kinase inhibitors
VEGFR: Receptor of vascular endothelial growth factor
